# Effects of Processing Parameters on the Microstructure and Mechanical Properties of Nanoscaled WC-10Co Cemented Carbide

**DOI:** 10.3390/ma15134472

**Published:** 2022-06-24

**Authors:** Yu Wang, Fengming Xiang, Xiaobo Yuan, Biaobiao Yang, Fenglin Wang, Yunping Li

**Affiliations:** 1 State Key Lab for Powder Metallurgy, Central South University, Changsha 410083, China; 203312134@csu.edu.cn (Y.W.); yuanxiaobo@csu.edu.cn (X.Y.); biaobiaoyang@csu.edu.cn (B.Y.); 2 Hong Shuo Intellgent Technology Co., Ltd., Jincheng 048000, China; csuxfm1301@163.com; 3 IMDEA Materials Institute, C/Eric Kandel 2, Getafe, 28906 Madrid, Spain; 4 Department of Materials Science, Polytechnic University of Madrid, E.T.S. de Ingenieros de Caminos, 28040 Madrid, Spain; 5 Candela (Shenzhen) New Energy Technology Co., Ltd., Shenzhen 518117, China; wangfenglin@ctiflywheel.com

**Keywords:** cemented carbide, mechanical properties, ball-milling time, sintering temperature, spark plasma sintering

## Abstract

This work was mainly focused on the processing-parameter-related microstructure and properties of ultrafine WC-10Co-0.4VC-0.5Cr_3_C_2_ cemented carbide. The samples were prepared via a spark plasma sintering (SPS) technique using nano WC and Co powders and the corresponding inhibitor VC and Cr_3_C_2_ powders. The influence of the processing process on the microstructure and mechanical properties of ultrafine-grained cemented carbide was investigated under different ball-milling times and sintering temperatures. The results showed that the grain size of WC decreased with increasing ball-milling time and decreasing sintering temperature and that the specific gravity of ε-Co increased with increasing ball-milling time. The hardness of cemented carbide increased with increasing ball-milling time and decreased with increasing sintering temperature due to the corresponding variation in grain size and the relative density of samples. The transverse fracture strength (TRS) was mainly affected by ball-milling time. The increase in ball-milling time led to decreased TRS values, mainly ascribed to the formation of WC particle agglomeration and the decreased WC-Co eutectic temperature. In addition, temperature changes were found to have little effect on TRS. The samples sintered at 1250 °C with a ball-milling time of 60 h had comprehensive mechanical properties. Their average grain size, relative density, hardness, and TRS were 355.5 nm, 95.79%, 2035.5 kg/mm^2^, and 2155.99 MPa, respectively.

## 1. Introduction

Cemented carbides are widely used in the automotive, aerospace, and military fields as an excellent cutting tool materials and mining material due to their high hardness, high strength and high wear resistance [1,2,3,4]. Since their wear resistance, strength and other properties are still stable at high temperatures, they are preferred in cutting tool materials in various fields and are popular in the manufacture of precision equipment. Cemented carbides are characterized by an insoluble hard compound (hard phase) as the matrix and a metal as the adhesive. With the ever-growing development of technology and the urgent demand in the field of precision machining, cemented carbide with superior mechanical performance is of great importance.

Generally, the grain size of WC is a key determinant of the mechanical performance of cemented carbide. Several studies have shown that the hardness, TRS, fracture toughness, and wear resistance of cemented-carbide-sintered samples can be significantly improved when the WC grain size is close to a sub-micrometer, especially under the nanocrystalline range (<0.5 µm) [3,5,6]. Therefore, controlling WC grain size is an important way to prepare high-performance ultrafine and nanocrystalline cemented carbide [7,8,9]. 

In recent decades, development of high-strength and high-hardness ultrafine-grained cemented carbides has become a worldwide challenge. Common strategies to refine the grain size of cemented carbide include adding metal carbides such as TiC, VC and Cr_3_C_2_ as grain-growth inhibitors (GGIs) [10,11,12] and/or regulating processing parameters using different sintering technologies [13]. The main role of GGI in refining grains is to inhibit the dissolution–reprecipitation process or the Ostwald ripening process [14], e.g., by forming a (W, V)C_x_ film at the interface for VC [15,16] and by dissolving it into the metal phase to increase the energy barrier of WC boundary movement for Cr_3_C_2_ [17,18]. Additionally, the superposition of different GGIs such as VC and Cr_3_C_2_ was found to produce synergistic effects to reduce grain size and contribute to improve mechanical properties [19]. Compared to traditional vacuum sintering (VS) and hot isostatic pressing (HIP) sintering, the spark plasma sintering (SPS) technique exhibits the lowest sintering temperature, the highest heating rate, and the lowest hold time, therefore best minimizing the possibility of grain size growth during sintering [20,21,22]. For instance, Huang et al. [23] reported that compared to the conventional liquid-phase sintering, cemented carbide prepared by SPS showed a reduced grain size and a higher Vickers hardness. Apart from sintering, ball milling and mechanical alloying also play important roles in affecting the grain size and comprehensive mechanical properties of cemented carbide. Several studies have shown that although the extension of ball-milling time increased the number of fine WC grains and reduced the eutectic temperature of WC-Co in the sintering process, particle agglomeration occurred after ball milling for a long time, thereby seriously affecting the mechanical properties [24,25,26]. It was reported that under heavy-duty ball-milling conditions, the properties of samples were severely affected, even with SPS sintering, due to the presence of grain agglomerates and microdefects [27]. In contrast, light ball milling can promote particle breakage and grain refinement, which are often correlated with improved mechanical properties. Moreover, with increases in ball-milling time, the grain size reached a saturated state and the degree of reduction was not obvious [28]. These results demonstrate that finding an optimum ball-milling time is necessary to obtain cemented carbide with the best mechanical properties. What is worse is that the combination of different parameters makes the selection of the best-parameter arrays more complicated; thus, few investigations in this field have been reported.

In this study, WC-10Co-0.4VC-0.5Cr_3_C_2_ cemented carbide was prepared via high-energy ball milling and SPS. The effects of different ball-milling processes (such as ball-milling time) and sintering temperatures on the microstructure and mechanical properties of ultrafine-grained cemented carbide were studied in detail.

## 2. Experiment

The target material of this experiment was WC-10wt.%Co-0.4 wt.%VC-0.5 wt.%Cr_3_C_2_ cemented carbide, which was mainly synthesized from WC powder and Co powder. The WC powder had an average grain size of 390 nm (Xiamen Tungsten Industry, Xiamen, China), as calculated with a Fischer particle size analyzer, and its specific surface area was 2.94 m^2^/g. The Co powder had an average grain size of 800 nm (Xiamen tungsten industry, Xiamen, China) and a specific surface area of 0.67 m^2^/g. The VC and Cr_3_C_2_ powder (ChaoWei Nanotechnology Inc., Shanghai, China) were added as GGIs. During processing, the raw powders and a certain amount of cemented carbide balls were placed into a cemented carbide ball-milling tank, and pure alcohol was added as the liquid medium. Then, these mixtures were ball-milled for 30 h, 60 h, and 90 h in a high-energy planetary mill (XH-XQM-4L). The corresponding parameters and symbols are shown in Table 1. The rotation speed and ball-to-powder ratio were 150 r/min and 5:1, respectively. Next, the powder mixture was dried in vacuum-drying oven (DZ-1BCⅡ) at 85 °C for several hours. Finally, these dried powders were sieved through 100 mesh sieves to prevent agglomeration. 

The milled powder mixture was placed in a graphite mold with a size of Φ 20.4 mm × 20 mm, and a pressure of 60 MPa was applied for 1 min. Then, the powder was sintered by SPS to obtain a sample with a pressure of 60 MPa. The powder sintering process adopted distributed gradient sintering, in which samples were heated to 1200 °C at heating rates of 100 °C/min, 150 °C/min, and 120 °C/min and then heated to temperatures of 1225 °C, 1250 °C, 1275 °C, and 1300 °C at a heating rate of 80 °C/min. Then, this temperature was maintained for 5 min before the samples were quickly cooled to room temperature. During the entire heating process, the pressure was slowly increased to 60 MPa from the start of sintering and then slowly decreased to normal pressure during cooling. 

The density of the samples was measured with Archimedes’ principle. The phase composition of the samples was analyzed with copper Kα radiation X-ray diffraction (XRD, D/max 2550 VB, Rigaku-Corporation, Tokyo, Japan) using fast and slow scans. The specific XRD equipment parameters were: a divergence slit of 1 deg, an anti-scatter slit of 1 deg, a receiving slit of 0.45 deg, a Sora slit of 0.3 mm, a graphite monochromator filter, a step size of 0.02 deg, a scanning speed of 8 deg/min, and a filament current of 450 mA. A field emission scanning electron microscope (SEM, Quanta 650 FEG, Prague, Czech Republic) was used to observe the microstructures and fracture morphologies of the prepared samples. For the SEM image of the surface of the polished samples, the image processing software Image-J (public image processing software based on Java) was used to measure more than 500 WC grains via the linear intercept method (ASTM-E112-13) in order to calculate the average grain size and grain cumulative distribution curve of each sample. Through the SEM energy dispersive spectrometer, the elemental distribution was systematically studied. TRS was measured with the three-point bending method (the alloy sample was placed on two fulcrums and a concentrated load was added to its midpoint). The samples were machined by wire EDM, and the cut surfaces were ground and polished to a surface roughness of Ra <= 0.4 µm with specimen dimensions of 16.5 mm × 6 mm × 5.2 mm (Model 3369 Table Mounted Materials Testing System, Instron-division of ITW LTD, Norwood, MA, USA). The spans and loading speed were 13 mm and 0.1 mm/min, respectively. The hardness of the sintered samples was measured with a Vickers hardness tester (mainly under a load of 5 kg), the loading and unloading duration was 15 s, and each sample were measured at least ten times to obtain the average value. 

## 3. Results and Discussion

### 3.1. Microstructure and Phase Constitution Analysis

**Figure 1:**
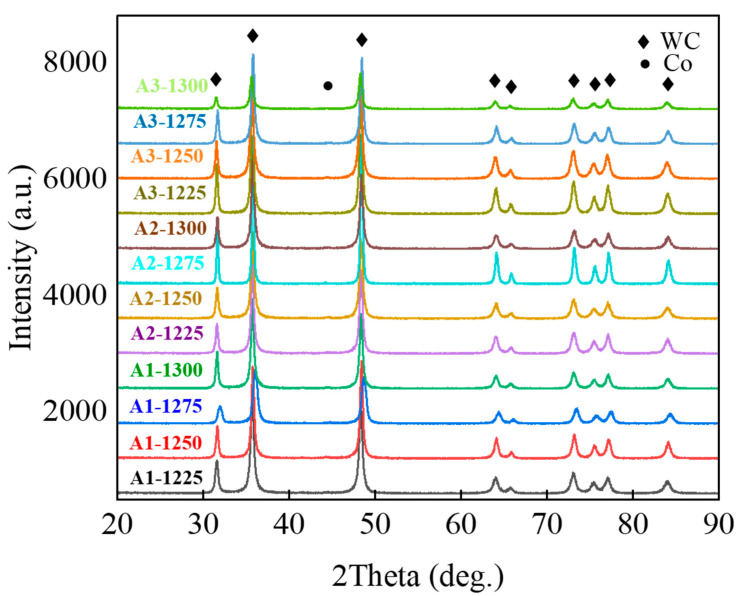
XRD patterns of sintered phases of WC-Co cemented carbides.

Figure 1 shows the XRD patterns of WC-10Co-0.4VC-0.5Cr_3_C_2_ cemented carbides under different ball-milling times and sintering temperatures. All samples were composed of WC and Co phases. No graphite phase was observed in all sintered samples. Two phases generally appeared in the absence of carbon: carbon-deficient (W_2_C) and η phases (Co_3_W_3_C and Co_6_W_6_C). These two phases harmed the mechanical properties of the cemented carbides [29]. As shown in Figure 1, we could not observe significant differences and the η phase was not observed in some sintered samples following increases in temperatures and ball-milling times. In addition, due to the relatively small contents of VC and Cr_3_C_2_, no peaks were observed in any alloy.

**Figure 2:**
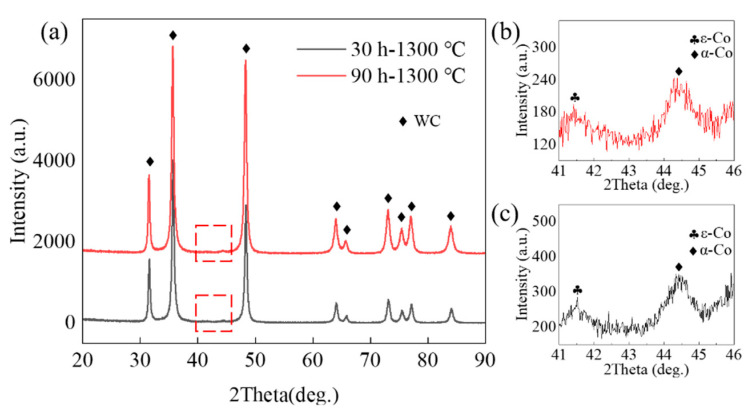
XRD patterns of (**a**) A1—1300 °C and A3—1300 °C WC-Co cemented carbide; (**b**) enlarged view of A1—1300 °C and (**c**) enlarged view of A3—1300 °C.

The XRD results of each samples showed little change at the macroscopic level, but there were some variations at the microscopic level. As shown in Figure 2a,b, it was found that in the range from 41° to 46°, there were two different Co phases, namely the α-Co and ε-Co phases. These two phases demonstrated significant influence on the mechanical properties of the cemented carbide. Among them, the existence of α-Co was conducive to the improvement of strength, and the transformation of α-Co into ε-Co negatively impacted strength [30]. For α-Co and ε-Co, the quantitative analysis results of the XRD adiabatic method at ball-milling times of 30 h and 90 h were as follows: the contents of α-Co were 54.8% and 45.2%, respectively, and the contents of ε-Co were 48.1% and 51.9%, respectively. It was found that the content of ε-Co increased with the increase in ball-milling time, demonstrating a serious effect on the subsequent TRS.

**Figure 3:**
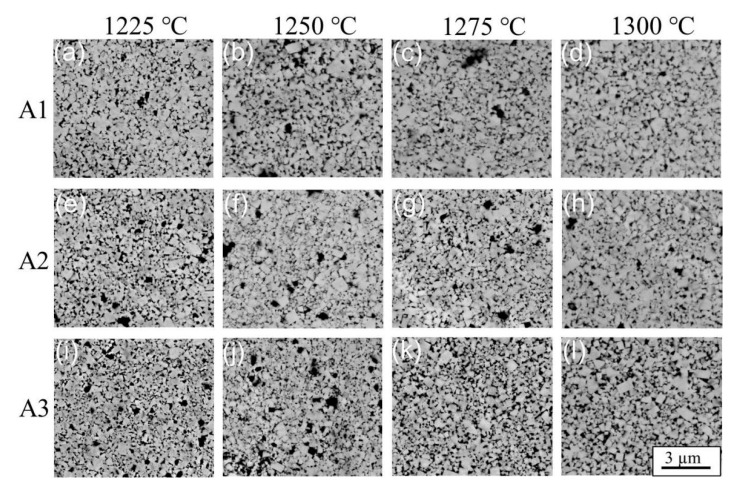
SEM (BSED) images of WC-Co cemented carbides under different sintering temperatures and ball-milling times: (**a**) A1—1225 °C; (**b**) A1—1250 °C; (**c**) A1—1275 °C; (**d**) A1—1300 °C; (**e**) A2—1225 °C; (**f**) A2—1250 °C; (**g**) A2—1275 °C; (**h**) A2—1300 °C; (**i**) A3—1225 °C; (**j**) A3—1250; °C (**k**) A3—1275 °C; and (**l**) A3—1300 °C.

Figure 3 shows SEM images of cemented carbides under different conditions, where the bright area represents WC phases, the dark area represents Co phases, and the black areas represent pores. It was found that Co was homogenously distributed around WC. Ascribed to the solid-state sintering and the lack of wettability between Co and WC, Co was not fully filled in the WC skeleton, resulting in the formation of a few pores and corresponding Co pools around them [31,32]. The appearance of Co pools increased the continuity of the WC/WC interface, which consequently increased the difficulty of crack propagation and affected properties such as TRS [31]. As shown in Figure 3a–d, it was obvious found that WC particles grew with the increase in temperature from 1225 °C to 1300 °C at the same ball-milling time of 30 h. As the sintering temperature rose, higher heat energy was generated, which provided greater driving force for Co and the dissolution–precipitation process. This led to the growth of WC grains. As shown in Figure 3a,e,i, the grain size of WC presented a clear trend of refinement with ball-milling times from 30 h to 90 h. As the ball-milling time increased, the frequency of the mixed powders colliding with each other under high-speed rotation increased. The same trend occurred at other temperatures and ball-milling times. Each WC particle obtained the approximate maximum kinetic energy, and these powders were more broken. Then, the number of finer WC particles increased. Therefore, the size of the obtained cemented carbide composite powder became smaller, and the WC grain size of the sintered sample accordingly decreased. In addition, no abnormally grown grains were found in all the images of cemented-carbide-sintered samples with different sintering temperatures and ball-milling times. 

**Figure 4:**
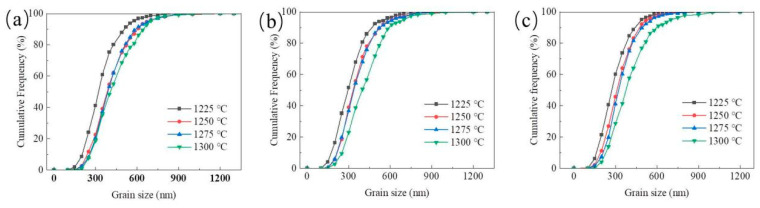
Cumulative distribution curve of the grain size of WC-Co cemented carbides under different ball-milling times: (**a**) A1, (**b**) A2, and (**c**) A3.

**Figure 5:**
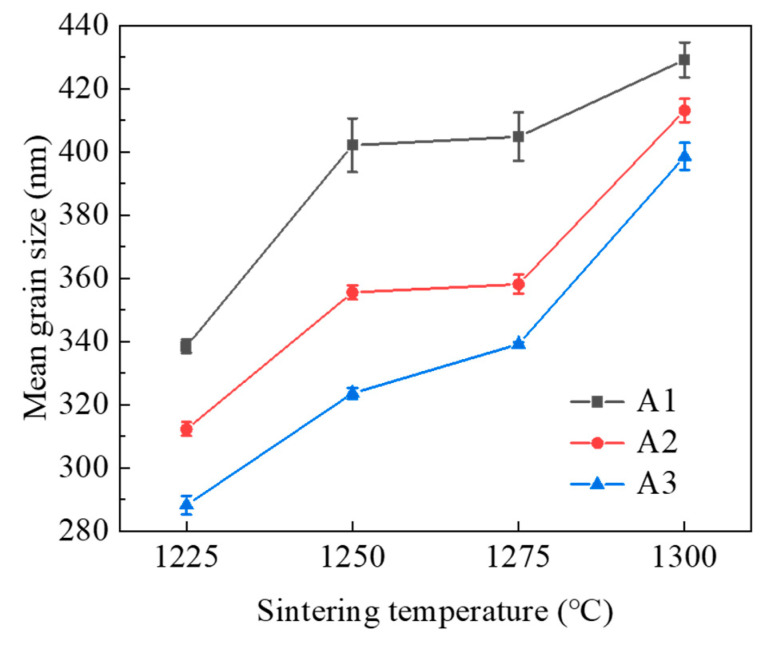
Average grain size of WC-Co cemented carbides at different ball-milling times and sintering temperatures.

Figure 4 and Figure 5 show the cumulative grain size distribution and mean grain size of sintered WC-Co cement carbides at different sintering temperatures and ball-milling times. As shown in Figure 4, it was found that with the decrease in sintering temperature, the grain size distribution became wider. This indicated that most of the grains were mainly concentrated in the small size range. As shown in Figure 4a, the cumulative grain size distribution of the cemented carbide samples tended to the left, and the grain size of about 41.2% of WC particles was less than 300 nm at 1225 °C. The grain size of the particles was less than 300 nm in about 21% of WC particles at 1250 °C and 1275 °C and about 18.7% of WC particles at 1300 °C. The same situation is also shown in Figure 4b,c. At the sintering temperatures of 1250 °C and 1275 °C, the cumulative grain size distribution curves of all ball-milling times were basically consistent and the average grain sizes were similar, indicating that the grain growth was not obvious in this temperature range. As shown in Figure 5, the average WC grain sizes at SPS sintering temperatures of 1300 °C, 1275 °C, 1250 °C, and 1225 °C were 429.12 nm, 404.77 nm, 402.12 nm and 338.47 nm, respectively, at 30 h of ball-milling time. The change of grain size between 1250 °C and 1275 °C was not obvious and similar to a bottleneck. Moreover, the grain size increased by 22% from 1225 °C to 1300 °C at 30 h of ball-milling time. Similarly, the grain size increased by 26% and 28% at ball-milling times of 60 h and 90 h, respectively. With the increase in ball-milling time, the crushing degree of WC particles intensified and finer cemented carbide particles were obtained. These fine WC particles had high surface activity, accelerating the dissolution and precipitation process of WC particles at higher temperatures and further improving the growth degree of particles in the sintering process. The results showed that the finer grain led to a greater the degree of grain growth at high temperatures. 

In addition, an interesting phenomenon was observed: compared to the increase in ball-milling time from 60 h to 90 h, the reduction in average grain size and the increase in the number of WC particles less than 300 nm were higher after milling time was increased from 30 h to 60 h. These results were more obvious at 1250 °C and 1275 °C: at 1250 °C, after the ball-milling time increased from 30 h to 90 h, the proportion of fine grains (grain size of less than 300 nm) increased from 22.6% to 39% and then to 45.8%. The increase rate of the 30 h of ball-milling time was 72%, which was much higher than 17.4% of the 90 h of ball-milling time. The same situation also occurred at 1275 °C. Although the degrees of change were small at 1225 °C and 1300 °C, the overall trend was still increasing. Due to the near equilibrium of crushing and cold welding, the finer powder was difficult to further refine and the overall particle size tended towards a saturated state [33]. This showed that after ball milling for 60 h, the size of WC particles reached a saturated state and the decrease in grain size was not obvious. At the same time, a large number of fine grains agglomerated, affecting the density of compacts and agglomerates. The results indicated that the overall powder was more fully refined after ball milling for 60 h. Grains with smaller sizes were obtained after 90 h, but the agglomeration seriously damaged mechanical properties.

### 3.2. Impact of Processing Parameters on Mechanical Properties 

**Figure 6:**
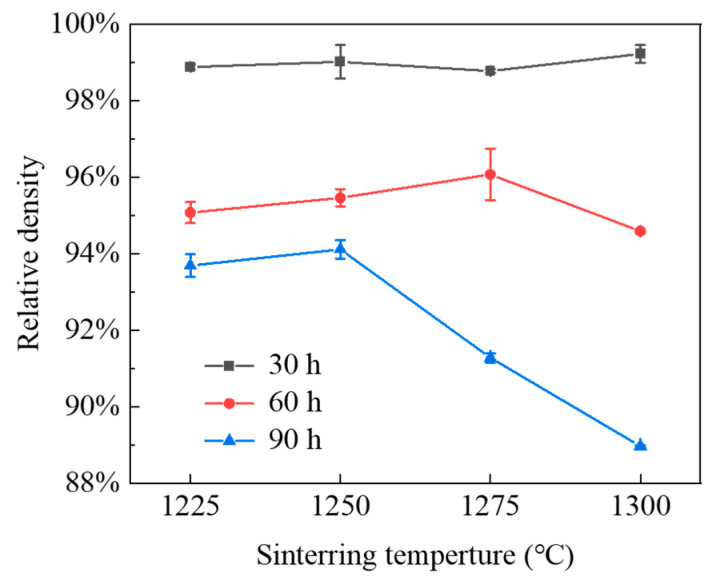
Relative density of WC-Co cemented carbide at different ball-milling times and sintering temperatures.

Figure 6 shows the relative density of WC-Co cement-carbide-sintered samples at different sintering temperatures and ball-milling times. It was found that the relative density of all samples was more than 98% after ball milling for 30 h, and the change of relative density was not obvious with changes of temperature. The same was true for a ball-milling time of 60 h, after which the relative density was around 95.5%. When the sintering temperature reached 1275 °C, the relative density of sintered sample reached 96.9%. This was mainly related to the dissolution of hard phase WC grains in Co. With the increase in temperature, the solubility of WC (there are solubility limits for W and C in liquid Co) increased and the WC grains dissolved in Co increased, which promoted the dissolution–precipitation process. In addition, the increase in temperature also provided heat energy, increasing the activity of Co and improving its driving force. Therefore, increasing its capillary force with WC grains enhanced the fluidity of the liquid phase to effectively fill the pores in the WC framework and slightly increased the density of the sample. However, when the eutectic temperature of the WC-Co composite powder was higher than 1300 °C, less of the liquid phase was produced. Therefore, it was still in the range of solid-state sintering, and the change of temperature had little effect on the relative density.

For the powder milled for 90 h, the relative density of the sample rapidly decreased. When the temperature increased from 1250 °C to 1275 °C, the density decreased from 94.61% to 91.26% and even reached 89% at 1300 °C. In the sintering process of cemented carbide, the main densification mechanisms were the rearrangement of WC particles, diffusion enhancement, and viscous flow of Co. In general, fine WC particles filled between coarse WC particles to increase their density and reduce the thickness of Co, thereby increasing the coverage and density of Co on the WC surface [34]. In addition, the eutectic temperature of WC-Co decreased due to the high-volume fraction of the finer nano powder surface and interface [28]. Compared to ball milling for 60 h, WC grains after milling for 90 h were finer, resulting in a decrease in the WC-Co eutectic temperature. When the temperature exceeded 1275 °C, pores appeared due to the dissolution loss of Co. With the increase in temperature, the loss increased and the relative density sharply decreased. In addition, fine WC grains agglomerated and a large number of grains appeared. Due to the contact between these aggregates and the matrix, the WC grains were not close and cracks easily appeared, which seriously affected their compactness and led to a reduction in relative density [34].

**Figure 7:**
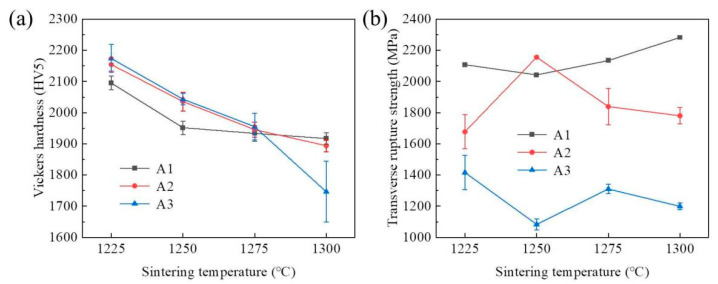
(**a**) Hardness and (**b**) transverse breaking strength of WC-Co cemented carbide at different ball-milling times and sintering temperatures.

Figure 7 shows the mechanical properties of WC-Co cement-carbide-sintered samples at the different sintering temperatures and ball-milling times. Figure 7a demonstrates that the Vickers hardness of the sintered samples generally reached the maximum after ball milling for 90 h. At the same sintering temperature of 1225 °C, the corresponding hardness values of the samples with ball-milling times of 90 h, 60 h, and 30 h were 2173.8 kg/mm^2^, 2154.5 kg/mm^2^, and 2095.1 kg/mm^2^, respectively. According to the Hall–Petch relationship, the size of WC grains has a great influence on hardness [35]. With the increase in temperature, the WC grains of each ball-milling time showed obvious coarsening phenomena. Then, their hardness decreased by 10%, 9.7%, and 7.7%, respectively, at 1225 °C, 1250 °C, and 1275 °C. When the sintering temperature reached 1300 °C, the hardness of the samples sintered for 90 h sharply decreased by about 11%. For 30 h and 60 h, the hardness was relatively stable at 1917 kg/mm^2^ and 1894.2 kg/mm^2^, respectively. The rapid decrease in hardness mainly occurred because the relative density of the sintered samples at 90 h was lower than that of other samples. In general, two important factors change the hardness of the sintered compacts: relative density and grain size. The former affects hardness due to the existence of pores, and the latter worsens the hardness due to grain coarsening [34,36,37]. When the sintering temperature was lower than 1300 °C, the decrease in its hardness was mainly caused by the coarsening of its grains, and the effect of relative density was not obvious. However, when the sintering temperature was as high as 1300 °C, the relative density sharply dropped, as shown in Figure 6. The main reason for this drop was that the combination of the agglomerates formed by the fine WC grains and the matrix was unstable, thus becoming the source of cracks and causing pores that had an important influence on the hardness. These results reveal that the relative density was the most important factor affecting hardness, as a rapid decrease in the relative density led to a rapid decrease in hardness. 

Figure 7b shows the TRS of cement carbide at various sintering temperatures under different ball-milling times. It was found that the change of TRS of sintered samples with sintering temperature was not obvious, but the corresponding TRS value decreased with increasing ball-milling times. When the sintering temperature reached 1225 °C, the TRS value decreased by about 20.4% (from 2107.7 MPa to 1677.26 MPa) from 30 h to 60 h and 15.6% (from 1677.26 MPa to 1416.105 MPa) from 60 h to 90 h. The fluctuation of TRS was about 5.6% (2107.7 MPa → 2042.21 MPa → 2134.44 MPa → 2282.28 MPa) with the change of temperature (1225 °C → 1250 °C → 1275 °C → 1300 °C) under the ball-milling time of 30 h. Similarly, for the ball-milling time of 90 h, the fluctuation of TRS was 13.3% (1416.11 MPa → 1083.32 MPa → 1310.33 MPa → 1198.97 MPa) with the change of temperature (1225 °C → 1250 °C → 1275 °C → 1300 °C). The overall change was not obvious. Only at the sintering temperature of 1250 °C for 60 h did the value of TRS reach 2155.99 MPa, which exceeded the 2042.21 MPa reached under the ball-milling time of 30 h, an increase of 28.5% compared to 1225 °C. 

**Figure 8:**
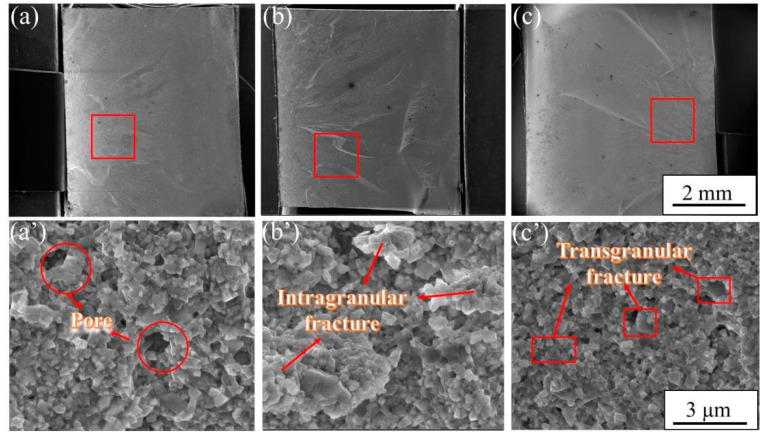
SEM (BSED) images of the fractured surfaces of the three samples at 1275 °C of (**a**) 90 h, (**b**) 30 h, and (**c**) 60 h; the red frames in (**a**–**c**) are corresponding high-resolution BSED images in (**a’**–**c’**).

To further clarify the aforementioned results, the surface morphologies of samples after fracture were investigated. Previous studies have shown that the TRS of sintered samples is related to many factors. It was reported that during SPS sintering, due to the poor fluidity of Co, agglomeration phenomena such as Co pooling can occur and seriously endanger the TRS [13]. In addition, in the absence of pores, TRS is inversely proportional to grain size, which is mainly related to the interfacial volume fraction of WC/Co [38]. Figure 8 shows the fracture surface and crack growth regions of WC-Co cemented carbides under three different conditions. It was found that WC-Co cemented carbide mainly presented brittle fracture, and its main crack-propagation modes were transgranular and intergranular fractures. The fracture surface was divided into two parts: a crack-propagation region with dense cracks and a fast fracture region with sparse cracks [39]. Figure 8a–c clearly shows that there were large differences in fracture morphologies of cemented carbides under different ball-milling times. For example, it was found that the fracture surface was relatively flat when milling for 90 h (Figure 8a). However, the crack growth regions under 30 h of ball-milling time were larger than those under 60 h of ball-milling time. The enlarged view of the local crack growth regions clearly shows the existence of pores at 90 h. The existence of pores was mainly caused by the insufficient fluidity of Co. Because the eutectic temperature of WC-Co was not reached in the sintering process, Co was not fully filled into the WC skeleton and the density of cemented carbide was not further improved. Therefore, these pores became fracture sources in the fracture process, which led to significant reductions in strength and hardness. Moreover, it can be seen from Figure 3 that under 90 h of ball-milling time, the agglomerates formed by the presence of many fine WC particles were not uniformly combined with the matrix and crack sources were easily formed under the action of external stress.

In addition to the influence of density difference on strength under 30 h and 60 h of ball-milling time, the WC/WC continuity factor is also worthy of consideration (Figure 3d,h). Generally, when the stress was sufficient, the squeezing stress acted on the hard phase WC, resulting in the brittle and transgranular fracture of WC grains. A ductile Co phase well-buffered the squeezing stress, in which good plasticity changed its fracture mode. The crack growth mode was mainly intergranular fracture moving along the WC/Co and WC/WC interfaces, decreasing the proportion of transgranular fractures. Therefore, the performance of TRS as greatly improved. Figure 8b,c clearly shows that there were a large number of rock candy crystals in the crack-propagation regions at 30 h, which was a typical intergranular fracture phenomenon. Many flat WC grains were found in the crack-propagation regions after ball milling for less than 60 h, which was a typical transgranular fracture; rock candy crystals were additionally observed. Compared to that of the intergranular fracture, the proportion was relatively small. In addition, the Co loss of finer powder was more serious during sintering at the same temperature. This directly affected the volume fraction of the WC/Co interface, resulting in a decrease in the buffer effect of Co. Therefore, the TRS at 60 h was slightly lower than that at 30 h (2134.44 MPa).

## 4. Conclusions

In this study, the effects of ball milling and sintering temperature on the microstructure and property of ultrafine-grained WC-Co cemented carbide were investigated. The main conclusions can be drawn as follows:

(1) The average grain size of WC-Co cemented carbide decreased with the increase in ball-milling time and coarsened with the increase in sintering temperature. The finest grain size of 288.22 nm was obtained after 90 h of ball milling and at a 1225 °C sintering temperature.
(2) Due to the Hall–Petch relationship, the hardness of sintered samples showed an opposite trend with the change of grain size, increasing with the decrease in grain size. The highest hardness of 2173.831 kg/mm^2^ was obtained after ball milling for 90 h and sintering at 1225 °C.
(3) With the increase in ball-milling time, the degree of the breakage and refinement of the WC particles was intensified. After 90 h, these refined WC particles agglomerated, which were unstable wen matching with the matrix. In turn, the relative density decreased and the hardness sharply dropped to 1746.25 kg/mm^2^ at 1300 °C. The results showed that the effect of relative density on hardness was greater than that of grain size.
(4) Compared to the results under ball-milling times of 30 h and 60 h, the grain size after 90 h was smaller, the eutectic temperature was the lowest, and the loss of Co was greater, thus resulting in the lowest TRS value.
(5) After 90 h of ball milling, there were obvious pores in the crack growth zone and the crack source was formed, which seriously endangered the mechanical properties. Furthermore, the increasing degree of transformation from α-Co to ε-Co and the increasing number of WC/WC interfaces also endangered the TRS of cemented carbide.

## Figures and Tables

**Table 1:** Ball-milling process and symbols for cemented carbides.

Symbol	Ball-Milling Time/h	Rotation Speed/rpm
A1	30	150
A2	60	150
A3	90	150

## Data Availability

Data available on request from the authors.
